# Targeting ferroptosis as a vulnerability in pulmonary diseases

**DOI:** 10.1038/s41419-022-05070-7

**Published:** 2022-07-26

**Authors:** Li Yang, Li-mian Cao, Xiao-ju Zhang, Bo Chu

**Affiliations:** 1 Department of Respiratory and Critical Care Medicine, Zhengzhou University People’s Hospital, Henan Provincial People’s Hospital, Zhengzhou, Henan China; 2 Department of Critical Care Medicine, The First Affiliated Hospital of Anhui Medical University, Hefei, Anhui China; 3 Department of Cell Biology, School of Basic Medical Sciences, Cheeloo College of Medicine, Shandong University, Jinan, Shandong China

**Keywords:** Cell death, Respiratory tract diseases

## Abstract

Ferroptosis is an iron-dependent regulated cell death marked by excessive oxidative phospholipids (PLs). The polyunsaturated fatty acids-containing phospholipids (PUFA-PLs) are highly susceptible to lipid peroxidation under oxidative stress. Numerous pulmonary diseases occurrences and degenerative pathologies are driven by ferroptosis. This review discusses the role of ferroptosis in the pathogenesis of pulmonary diseases including asthma, lung injury, lung cancer, fibrotic lung diseases, and pulmonary infection. Additionally, it is proposed that targeting ferroptosis is a potential treatment for pulmonary diseases, particularly drug-resistant lung cancer or antibiotic-resistant pulmonary infection, and reduces treatment-related adverse events.

## FACTS

- Ferroptosis is a regulated cell death induced by iron-driven lipid peroxidation.
- Different pathways participate in ferroptosis induction and defense.
- Ferroptosis is closely related to the development of pulmonary diseases and acts as a potent choice for pulmonary diseases treatment.

## Open questions

- What is the regulation progress of iron metabolism in the cell?
- How is lipid peroxidation produced and eliminated by anti-ferroptosis pathways in the regulation of ferroptosis?
- How does ferroptosis participate in the regulation of pulmonary diseases pathophysiology and therapy choices?

## Introduction

In 2012, Brent R. Stockwell et al. identified two structurally unrelated small molecules (erastin and RSL-3) named RAS-selective lethal (RSL) compounds, which selectively kill oncogenic RAS mutant cell lines [1, 2]. The RSL compounds-induced cell death is associated with increased peroxidized lipids and could be prevented by iron chelator deferiprone or deferoxamine (DFO), which is different from apoptosis, necrosis, or other types of regulated cell death [2–4]. This iron-dependent non-apoptotic induced cell death is named ferroptosis [5]. Subsequently, they identified ferrostatin-1 (Fer-1) and liproxstatin-1 (Lip-1) as specific inhibitors of ferroptosis [5, 6].

Ferroptosis, associated with two major biochemical features: iron accumulation and lipid peroxidation, is a reactive oxygen species (ROS) dependent form of cell death [7]. Molecules of free radicals with unpaired electrons in their outer shells, including superoxide radical (O_2_^-^), hydrogen peroxide (H_2_O_2_), and the hydroxyl radical (HO·) produced in autoxidation reactions are important components of ROS in the cell [8]. The production of ROS contributes to DNA damage, metabolic reprogramming, lipid peroxidation, and eventually ferroptosis [9–11]. Mitochondrial morphology is also identified as a marker of ferroptosis execution [5]. Morphological changes of mitochondria are observed in ferroptosis as condensation or swelling, increased membrane density, decreased crista, and ruptured outer membrane with an electron microscope [12].

**Fig. 1:**
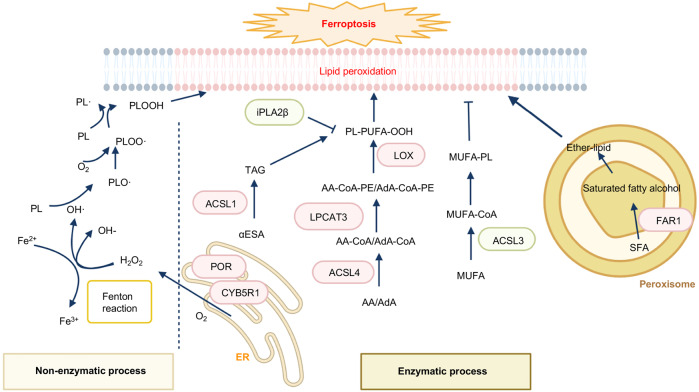
The lipid peroxidation associated pathways. The non-enzymatic process (as Fenton reaction) and enzymatic process (as ACSL4, LPCAT3, LOXs, FAR1, POR) participate in ferroptosis by the production of lipid peroxidation in membrane PLs. The enzyme-induced ferroptosis is colored in a red box, while the enzyme-inhibiting ferroptosis is colored in a green box.

**Fig. 2:**
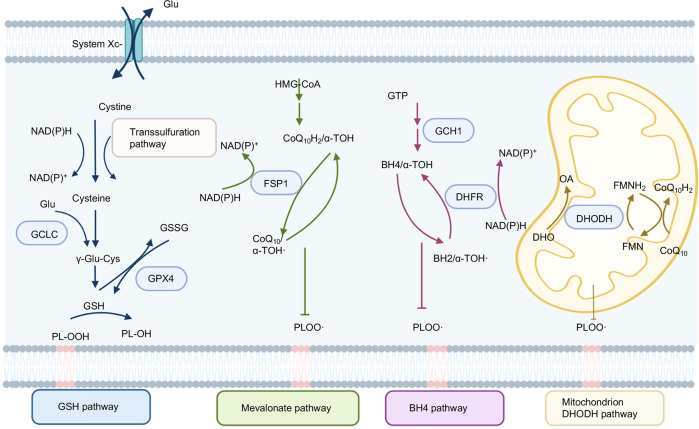
Ferroptosis defense pathways. The related genes are marked in a blue box. The GSH pathway contains GSH synthesis (GCLC, trans-sulfuration pathway), Glu exporter (System Xc-), and GSH reduction (GPX4). The mevalonate pathway is mainly involved in FSP1-mediated CoQ reduction. BH4 pathway contains GCH1-mediated BH2 synthesis and DHFR-stimulated conversion of BH2 to BH4. DHODH is functional in the mitochondrion.

Membrane-bound polyunsaturated lipids containing bis-allylic carbons are highly susceptible to lipid peroxidation under the presence of oxygen [13]. Both the non-enzymatic process and enzymatic production of phospholipid hydroperoxides (PLOOH) are iron-dependent [14]. The non-enzymatic process utilizes iron and oxygen to catalyze the formation of PLOOH via the Fenton reaction (Fig. 1) [15]. Fenton chemistry catalyzed by Fe^2+^, instead of copper (Cu^2+^), zinc (Zn^2+^), and cobalt (Co^2+^), converting peroxides into free radical productions and lipid peroxidation is the major cause of ferroptosis [5]. In the enzymatic process, the lipoxygenases (LOXs or ALOXs) and NADPH-cytochrome P450 reductase (POR) are implicated to generate PLOOH [14]. Once PLOOH is not rapidly cleared by free radical scavengers or defending pathways including GPX4-GSH, FSP1-COQ_10_, DHODH-COQ_10,_ and GCH1-BH4 (Fig. 2), the chain reaction occurs: PUFA-PLs react with cellular labile iron to generate alkoxyl and peroxyl radicals, then further propagating PLOOH productions [16]. Therefore, it is an effective approach to verify lipid peroxidation and ferroptosis by utilizing probes (C11-BODIPY 581/591) to directly detect oxidized PUFA-PLs [5]. Moreover, reactive toxic aldehydes among lipid peroxidation products such as 4-hydroxy-2-nonenal (4-HNE) or malondialdehyde (MDA) are confirmed as markers of oxidative stress-induced lipid peroxidation [17, 18].

The lung is the organ with the largest surface area in the human body, representing a unique interface with the outside environment, exchanging oxygen and carbon dioxide, and susceptible to damage from inflammatory responses [19, 20]. Exposing to the outside environment, the nonpathogenic antigen activates inflammatory cells to lead to intracellular oxidative damage in respiratory mucosal surfaces [21], which contributes to respiratory illnesses [22]. Furthermore, noxious particles or gases, mostly cigarette smoking (CS) induced ROS production accompanied by ferroptosis are related to lung diseases, such as chronic obstructive pulmonary disease (COPD), lung cancer, pulmonary infections, and acute lung injury [23, 24].

Overall, the generation of iron-induced lipid peroxidation, and reduction of oxidized peroxides via anti-ferroptosis pathways are important to maintain cell homeostasis and influence the progression of pulmonary disease.

## Iron homeostasis

**Fig. 3:**
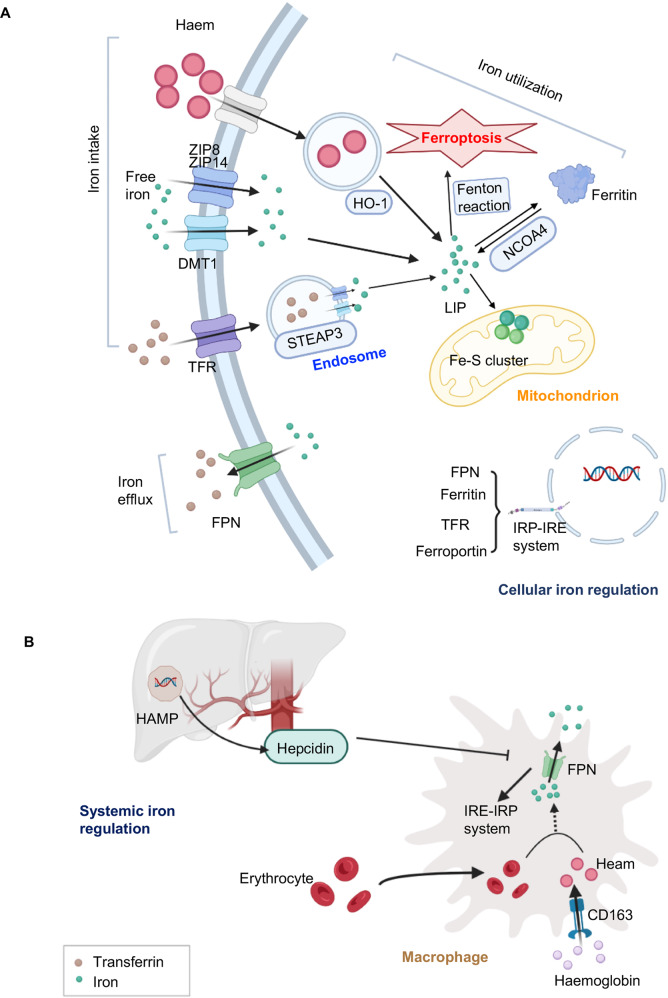
Iron metabolism. **A** The regulation of system iron. Senescent erythrocytes are swallowed by macrophages to release iron via FPN to the system iron pool. Then hemoglobin is combined with haptoglobin endocytosis by the scavenger receptor CD163 on macrophages to release heme utilized in the cell. Hepcidin, encoded by HAMP in hepatocytes, is secreted to regulate the expression of FPN to maintain the system iron level. **B** Transferrin, heme, or free iron is transported into cells through different transporters. Transferrin or heme is internalized by the endosome and the free ferrous iron is directly stored at LIP or synthetic ferritin store. The mitochondrion is also an important site to utilize iron to synthesize Fe-S clusters. FPN is a unique pathway known until now.

Iron is required for ROS production, changing cellular labile iron pool (LIP) content, and altering the sensitivity of ferroptosis [14]. The uptake of iron (transferrin (TF)), non-transferrin-bound iron (free iron), heam, and hemoglobin, storage (ferritin), utilization (nuclear receptor coactivator 4 (NCOA4)) or efflux (solute carrier family 40 member 1 (SLC40A1/ferroportin-1 (FPN))) are important to maintaining intracellular iron homeostasis (Fig. 3A). For iron transport, TF binds with serum Fe^3+^, and the transferrin receptor (TFR) within the membrane recognizes TF to facilitate iron uptake in an endosome-dependent manner [25]. The six-transmembrane epithelial antigen of prostate family member 3 (STEAP3), a metal reductase, reduces Fe^3+^ to Fe^2+^ in the endosome, ultimately releasing free iron into the cytosol. Subsequently, membrane metal-iron transporters divalent metal-ion transporter 1 (DMT1), zrt- and irt-like protein 14 (ZIP14), and ZIP8 transport free iron to the cytoplasm [7, 26]. Heme oxygenase 1 (HMOX-1/HO-1), a well-known antioxidant enzyme, initiates heme degradation to release iron [27]. Excess labile iron is stored by ferritin, whereas NCOA4 facilitates ferritin-bound iron into the cytosol via ferritinophagy-mediated degradation of ferritin (Fig. 3A) [28, 29]. Besides, FPN, the only cellular iron efflux transporter identified, is engaged in extruding iron into the extracellular space [30].

Iron metabolism is mainly mediated by hepcidin and iron-responsive element (IRE) - iron-regulatory protein (IRP) system-regulated cellular signaling pathways (Fig. 3B) [31, 32].

Hepcidin is a regulatory hormone secreted by hepatocytes that controls plasma iron levels via modulating dietary iron absorption, circulating hemoglobin iron release, and stored iron movement [33]. Hepcidin decreases blood iron by binding with SLC40A1, thereafter triggering SLC40A1 internalization and degradation to reduce iron release to serum. On the other hand, SLC40A1 -mediated iron export decreases the cellular iron level, which induces the expression of IRP due to iron starvation [34]. Activated IRPs (IRP1 and IRP2) consequently bind with IREs of ferritin and SLC40A1 to inhibit their translations and stabilize expressions of TFR, therefore restoring labile iron homeostasis [32, 35, 36]. Then, IRPs disassociate from IREs in iron-replete cells and undergo iron-dependent degradation.

The crosstalk between hepcidin-SLC40A1 mediated system iron metabolism and IRP-IRE regulated cellular iron homeostasis is that the IRP-IRE system senses cellular iron starvation and limits iron losses, whereas hepcidin-FPN protects the organism against systemic iron overload. Besides, IRP-IRE regulates hepcidin through the expression of TFR, which is a signal to modulate the level of hepcidin [31]

## Lipid peroxidation

Lipid peroxidation of the membrane polyunsaturated fatty acids (PUFAs) is the hallmark of ferroptosis [14]. Depending on the double bond position of the methyl terminal (ω; n-) end, PUFAs are classified into Omega 6 (n-6) and Omega 3 (n-3) respectively. N-6 or n-3 PUFAs are essential fatty acids for human beings and are mainly taken up from the diet [37]. The accumulation of n-3 or n-6 PUFAs is toxic and selectively induced ferroptosis in cancer cells under ambient acidosis in vitro. Furthermore, the n-3 PUFAs-rich diet significantly delays tumor growth through ferroptosis in mouse models [38].

PLs are the fundamental constituents of biological membranes. In mammals, glycerophospholipids are the major PLs [39]. To be more specific, phosphatidylethanolamine (PE) is the key inducer of ferroptosis [40]. During the occurrence of ferroptosis, free PUFAs esterified to acyl-CoA, membrane phospholipid remodeling, followed by ALOXs/LOXs-stimulated oxidation are indispensable for membrane lipid peroxidation in an enzyme-dependent way [14] (Fig. 1). The associated enzymes are discussed as follows and the related genes are listed in Table 1.

**Table 1:** Functions of lipid peroxidation-related genes and their promotion or resistance of ferroptosis.

Gene	Function	Promote or resist ferroptosis	Reference(s)
ACSL4	Addition of CoA to free long-chain PUFAs	Promote	[41]
ACSL3	Catalyze MUFAs to fatty acyl-CoAs	resist	[44]
ACSL1	Accumulation of αESA in TAGs	Promote	[43]
LPCAT3	Catalyzes the insertion of acylated AA into membrane phospholipids	Promote	[47]
ALOX	Enhance AA- or AdA-containing diacylated PEs production	Promote	[50, 51]
FAR1	Converting SFA to fatty alcohol to synthesized PUFA-ePLs in Peroxisomes	Promote	[55, 56]
iPLA2β	Eliminate peroxidized arachidonoyl-PE species	resist	[48, 49]
POR and CYB5R1	Transfer electrons from NAD(P)H to oxygen to generate H_2_O_2_ to induce Fenton reaction and ferroptosis	Promote	[57, 58]

*ACSL4* Long-chain acyl-coenzyme A synthase 4, *ACSL3* Long-chain acyl-coenzyme A synthase 3, *ACSL1* Long-chain acyl-coenzyme A synthase 1, *PUFAs* polyunsaturated fatty acids, *MUFAs* monounsaturated fatty acids, *LPCAT3* lysophosphatidylcholine acyltransferase 3, *AA* arachidonic acid, *AdA* adrenic acid, *αESA* α-eleostearic acid, *TAGs* triacylglycerols, *ALOX* lipoxygenase, *FAR1* Fatty acyl-CoA reductase 1, *SFA* saturated fatty acid, *PUFA-ePLs* polyunsaturated ether phospholipids, *iPLA2β* Ca2+-independent phospholipase A2β, *PE* phosphatidylethanolamine, *POR* cytochrome P450 oxidoreductase, *CYB5R1* cytochrome b5 reductase.

### Free fatty acid esterification

The long-chain acyl-CoA synthases (ACSLs) family has five isoforms: ACSL1, ACSL3, ACSL4, ACSL5, and ACSL6. Among ACSLs, acyl-CoA synthetase long-chain family member 4 (ACSL4) is the first one identified promoting lipid peroxidation [41]. ACSL4 is responsible for the addition of CoA to free long-chain PUFAs, especially the synthesis of PE-containing arachidonic acid (AA)-CoA or adrenic acid (AdA)-CoA [40].

Besides, ACSL1 is recently found essential for α-eleostearic acid (αESA) induced ferroptosis. ACSL1 promotes the production of neutral lipids, such as diacylglycerols (DAGs) and triacylglycerols (TAGs) [42], and sensitizes cells to ferroptosis by accumulating αESA in TAGs. By supplementing with tung oil, a rich source of αESA, tumor cell ferroptosis induced in vitro, tumor growth and metastasis are suppressed in mice [43].

By contrast, ACSL3 is an important ferroptosis-resistant gene replacing PUFAs in the PLs with monounsaturated fatty acids (MUFAs) [44]. The disorder of ACSL3 is related to poor survival in lung adenocarcinoma (LUAD) [45].

### Membrane phospholipid remodeling reactions

Deacylation and re-acylation reactions, called Lands’ cycle, maintain the diversity of fatty acyl composition of membranes PLs and sensitivity to ferroptosis [46]. Lysophosphatidylcholine acyltransferase 3 (LPCAT3), which is a member of acylation enzymes, preferentially catalyzes the insertion of acylated AA by ACSL4 into membrane PLs. The loss of LPCAT3 or ACSL4 depletes the substrates for lipid peroxidation and increases ferroptosis resistance [47].

Deacylation enzymes such as Ca^2+^-independent phospholipase A2β (iPLA2β) hydrolyzes acyl tails from the glycerol backbone of lipids and releases PUFA from PLs. Meanwhile, iPLA2β preferentially hydrolyzes peroxidized PLs, thereby eliminating peroxidized arachidonoyl-PE species produced by ALOXs family members to defend ferroptosis [48]. Furthermore, iPLA2β is sufficient to suppress p53-induced ferroptosis upon ROS stress in a glutathione peroxidase 4 (GPX4) independent way. The loss of iPLA2β, in contrast to GPX4 (which will be discussed below), has no influence on the normal development or cell viability in normal tissues, whereas iPLA2β plays an essential role in ROS-induced ferroptosis in tumor cells [49]. Indeed, iPLA2β-/- mice develop normally while systematic deletion of GPX4 results in embryonic lethality. Therefore, it is proposed that iPLA2β is a more promising therapeutic target compared with GPX4 for ferroptosis-targeted therapy in human cancers without causing severe toxicity.

### ALOXs/LOXs -stimulated lipid oxidation

ALOXs/LOXs are non-heme iron-dependent dioxygenases that catalyze free PUFA oxygenation or PUFA-containing lipids oxidation in biological membranes to promote ferroptosis [50, 51]. There are six functional LOX genes (ALOX15, ALOX15B, ALOX12, ALOX12B, ALOXE3, ALOX5) in humans, which encode for six different ALOX isoforms and digits indicate oxygens introduced at the number of the carbon atom of the arachidonic acid backbone [52]. Among the ALOX isoform, overexpression of ALOX5, ALOX12, and ALOX15 significantly sensitize cells to ferroptosis [50]. Additionally, ALOX12 or ALOX15 directly oxygenates AA containing PLs, while other LOX isoforms need cytosolic PLA2 prior to hydrolysis of esterified AA from PLs [53].

The enzymes (ACSL4, LPCAT3, ALOX15) are determinants of PLOOH production. In addition, ALOX12 is essential in p53-mediated ferroptosis, which is independent of the ACSL4-GPX4 pathway [54], indicating a new potential approach for tumor treatments.

### Peroxisome-ether-lipid axis

It is well established that long-chain PUFAs induce ferroptosis and MUFAs enhance ferroptosis resistance. Recent research reveals that long-chain saturated fatty acids (SFAs) also participate in ferroptosis [55, 56]. Fatty acyl-CoA reductase 1 (FAR1) is an essential factor for SFAs-mediated ferroptosis by converting SFAs to fatty alcohol, which is required for the synthesis of alkyl-ether lipids and plasmalogens (Fig. 1). Knockdown of key enzymes such as GNPAT or AGPS involved in ether lipid biosynthesis largely eliminates SFAs or FAR1 induced ferroptosis. The peroxisome is crucial for ether-lipid mediated ferroptosis, as depletion of peroxisomes by knocking out PEX genes remarkably decreased ether lipids synthesis and substantially blocked ferroptotic cell death [55, 56].

### ER-residing oxidoreductases

POR and NADH-Cytochrome B5 Reductase 1 (CYB5R1) are oxidoreductases resident in the endoplasmic reticulum (ER). These two enzymes produce H_2_O_2_ by transferring electrons from NAD(P)H to oxygen. Subsequently, generated H_2_O_2_ reacts with Fe^2+^ to induce PLOOH. Compared with POR, CYB5R1 has a lower ability to transfer electrons and produces H_2_O_2_-mediated lipid peroxidation. However, CYB5R1 synergizes with POR together to induce lipid peroxide formation and ferroptosis [57]. In addition, POR is expressed in most tissues, while ALOXs are expressed in limited tissues. The POR pathway induces lipid peroxidation and ferroptosis in the cells expressing low levels of ALOXs, indicating that targeting POR or CYB5R1 is liable to a more potential therapeutic approach [58].

## Ferroptosis defense pathways

### Protective mechanism against peroxidation damage

There are four major anti-ferroptosis pathways (Fig. 2), and the related genes protecting cells from ferroptosis are listed in Table 2. Cyst(e)ine/GSH/GPX4 axis is regarded as the mainstay in anti-ferroptosis progression.

**Table 2:** Cellular localization and function of ferroptosis resistance genes.

Gene	Function	Location	Reference
SLC7A11	Mediates the cystine/glutamate antiporter to GSH synthesis.	Membrane	[62]
GPX4	Converting peroxidized PUFA-containing PLs to non-toxic PL-OH.	Cytoplasm, mitochondrion	[64, 65]
FSP1	NAD(P)H-dependent CoQ oxidoreductase to suppress lipid peroxidation and ferroptosis via reducing CoQ to CoQH_2_.	Membrane	[67, 68]
DHODH	DHODH suppresses mitochondrial lipid peroxidation and ferroptosis in a CoQ-dependent way by converting CoQ to CoQH2	mitochondrion	[72]
DHFR	Mediated regeneration BH4 from BH2. BH4 availability might reduce CoQ to enhance ferroptosis resistance	Cytoplasm	[74]
GCH1	The rate-limiting enzyme for BH4 synthesis.	Cytoplasm	[75]

*SLC7A11* solute carrier family 7 member 11, *GSH* glutathione, GPX4 glutathione peroxidase 4, *FSP1* ferroptosis suppressor protein 1, *DHODH* dihydroorotate dehydrogenase, *DHFR* dihydrofolate reductase, *GCH1* guanosine triphosphate cyclohydrolase 1, *PUFA* polyunsaturated fatty acid, *PLs* phospholipids, *PL-OH* PL-alcohol, *CoQ* ubiquinone, *CoQH*_*2*_ ubiquinol, *BH4* tetrahydrobiopterin, *BH2* dihydrofolic acid.

### Cyst(e)ine/GSH/GPX4 axis

#### System Xc^−^

The System Xc^−^ is a transporter to import extracellular cystine and simultaneously export intracellular glutamate at a 1:1 ratio [59]. It consists of two subunits: the solute carrier family 7 member 11 (SLC7A11/ xCT) mediates the cystine/glutamate transport, and the solute carrier family 3 member 2 (SLC3A2) stabilizes SLC7A11 protein with appropriate membrane localization [60]. System Xc^-^-related cystine transportation is important to maintain intracellular cysteine levels as it is a critical precursor for glutathione (GSH) synthesis [61] serving as a co-factor for glutathione peroxidase 4 (GPX4) to detoxify lipid peroxides [12]. Erastin significantly blocks SLC7A11-mediated cystine uptake to limit GSH synthesis to induce ferroptosis [5]. Although the trans-sulfuration pathway utilizes methionine and serine to synthesize cellular cysteine in some tissues or cell lines to support the GSH synthesis, system Xc^-^ is more important to cysteine, as SLC7A11-KO cell lines need β-mercaptoethanol (β-ME) to promote cystine uptake through an alternative pathway [62, 63]. The loss of SLC7A11 substantially abolishes tumorigenesis while the overexpression of SLC7A11 promotes tumor development through ferroptosis resistance [62].

#### GPX4

In mammalian tissues, the major mechanism to detoxify H_2_O_2_-induced ROS is dependent on glutathione peroxidases (GPXs). All GPXs family members eliminate cytosolic ROS. However, GPX4, a selenoprotein containing selenocysteine (Sec) [64] distributing throughout the cytosol and mitochondria, possesses a unique catalytic capability to convert peroxidized PLs to non-toxic PLs-alcohol (PL-OH) using GSH as substrate [65].

### AIFM2/FSP1-CoQ axis

Apoptosis-inducing factor mitochondria-associated 2 (AIFM2) is a flavoprotein that was originally claimed to induce apoptosis [66]. AIFM2/FSP1 is identified as an important anti-ferroptosis gene utilizing CRISPR screen or cDNA library transduction [67, 68]. FSP1 renders cells resistant to ferroptosis independent of the GPX4-GSH pathway.

FSP1 is located on the lipid droplets or the plasma membrane but not on ER or mitochondria defined by fluorescence detection of the tagged genomic locus FSP1 [67]. Nevertheless, plasma-membrane localization of FSP1 is necessary and sufficient to protect against ferroptosis, whereas FSP1 located within the lipid droplets is not required for ferroptosis protection [67]. N terminus of FSP1 contains canonical myristoylation, which facilitates FSP1 interaction with lipid bilayers and is essential for defending ferroptotic cell death [68]. Mutation of the myristoylation site markedly weakens the FSP1-mediated anti-ferroptotic effect. FSP1 acts as a NAD(P)H-dependent ubiquinone (CoQ) oxidoreductase to suppress lipid peroxidation and ferroptosis via reducing CoQ to ubiquinol (CoQH_2_) [67, 69].

In lung cancer cell lines, the expression of FSP1 is negatively correlated with the sensitivity to RSL3, ML162, or ML210 (GPX4 inhibitors) [68]. FSP1 maintains the lung tumor growth of GPX4 knockout H460 cells in tumor xenograft mouse models under the treatment of IKE (System Xc- inhibitor and ferroptosis inducer in vivo) [68, 70]. Therefore, based on the characteristic of membrane location and strong protection against ferroptosis, FSP1 is a potential candidate for ferroptosis-targeted drug discovery.

In addition, emerging studies reveal that GPX4 inhibitors such as RSL-3 and ML162 efficiently suppress tumor growth, implying that GPX4 could be a promising target for tumor inhibition. However, these researches ignore the effect on surrounding normal tissues, as these compounds lack specific targeting to tumor cells. A recent study investigates systemic deletion of GPX4 in mice causing embryonic lethality [71]. Furthermore, inactivation of GPX4 in normal tissues induces severe acute tissue injury, which is partially rescued by deficiency of ALOX15 [6]. These findings suggest that GPX4 may serve as a ‘housekeeping’ gene to control the levels of lipid peroxidation in multiple kinds of cells or tissues. As the current GPX4 inhibitor could not specifically recognize tumor cells, future clinical study should focus more attention on the development of compounds specifically target to GPX4 of tumor cells.

### Mitochondria DHODH

Dihydroorotate dehydrogenase (DHODH), which participates in *de novo* pyrimidine synthesis, is located on the outer surface of the inner mitochondrial membrane [72]. In the cancer cells with low expression of GPX4, DHODH markedly protects cells from ferroptosis. Combined inhibition of mitochondria localized-GPX4 and DHODH strongly induces mitochondrial lipid peroxidation and rapid cell death. The mitochondria-localized GPX4, not cytosol-GPX4 coordinates resistance to ferroptosis with DHODH. Moreover, DHODH-mediated ferroptosis is independent of FSP1, as ectopic expression of mitochondria-localized FSP1 is unable to protect cells from ferroptosis [73]. DHODH suppresses mitochondrial lipid peroxidation and ferroptosis in a CoQ-dependent way by converting mitochondrial CoQ to CoQH_2_. The specific DHODH inhibitor brequinar selectively suppresses cancer cells with low expression of GPX4 induced tumor growth. Moreover, combined treatment with brequinar and sulfasalazine synergistically facilitates ferroptosis and efficiently abrogates tumor growth induced by cells with high expression of GPX4 [73].

### GCH1-BH4-phospholipid axis

Sustained cystine depletion and GPX4 inhibition impair cell proliferation via distinct mechanisms across different cell types [74]. Activation of guanosine triphosphate cyclohydrolase 1 (GCH1), the rate-limiting enzyme for tetrahydrobiopterin (BH4) synthesis, is a potent anti-ferroptosis approach in GPX4-inhibited cells. Although previous study implies that BH4 might enhance ferroptosis resistance by reducing CoQ to CoQH_2_ [75], later research demonstrates that BH4 protects cells against PLs peroxidation as a potent radical-trapping antioxidant independent of its co-factor role in GPX4 inhibition [74]. Supplementation of BH2 in vitro protects the cell from ferroptosis through dihydrofolate reductase (DHFR)-mediated regeneration BH4 [74]. Either BH4 alone or combined BH2 with DHFR showed superior inhibitory activity of lipid peroxidation than the individual component.

### NRF2-mitigated lipid peroxidation and ferroptosis

Nuclear factor erythroid 2-related factor 2 (NRF2) is a transcription factor that regulates cellular antioxidant response with a low basal expressed level during unstressed conditions in all most cell types [76]. KEAP1-NRF2 axis is the prominent regulatory pathway to keep the low expression of NRF2 through ubiquitylation and proteasomal degradation of NRF2 [77]. Under stress such as amino acid deprivation or oxidative condition, NRF2 migrates to the nucleus to initiate transcription of the antioxidant response element (ARE)-containing genes via disassociation from KEAP1 [78].

Besides of the regulation of iron metabolism, NRF2 regulates GSH synthesis to defend against ferroptosis via modulating the expression of SLC7A11 and the gamma-glutamylcysteine ligase (GCL) system. GCL contains two subunits, the glutamate-cysteine ligase catalytic subunit (GCLC) and modulatory (GCLM) [79], which is the rate-controlling enzyme participating in GSH synthesis. The basal or inducible expression of GCLC and GCLM is controlled by NRF2. In addition, targets of NRF2 (AKR1B1 and AKR1B10) regulate lipid metabolism by reducing aldehydes and ketones to less toxic alcohol forms [77].

## The role of ferroptosis in pulmonary diseases

**Fig. 4:**
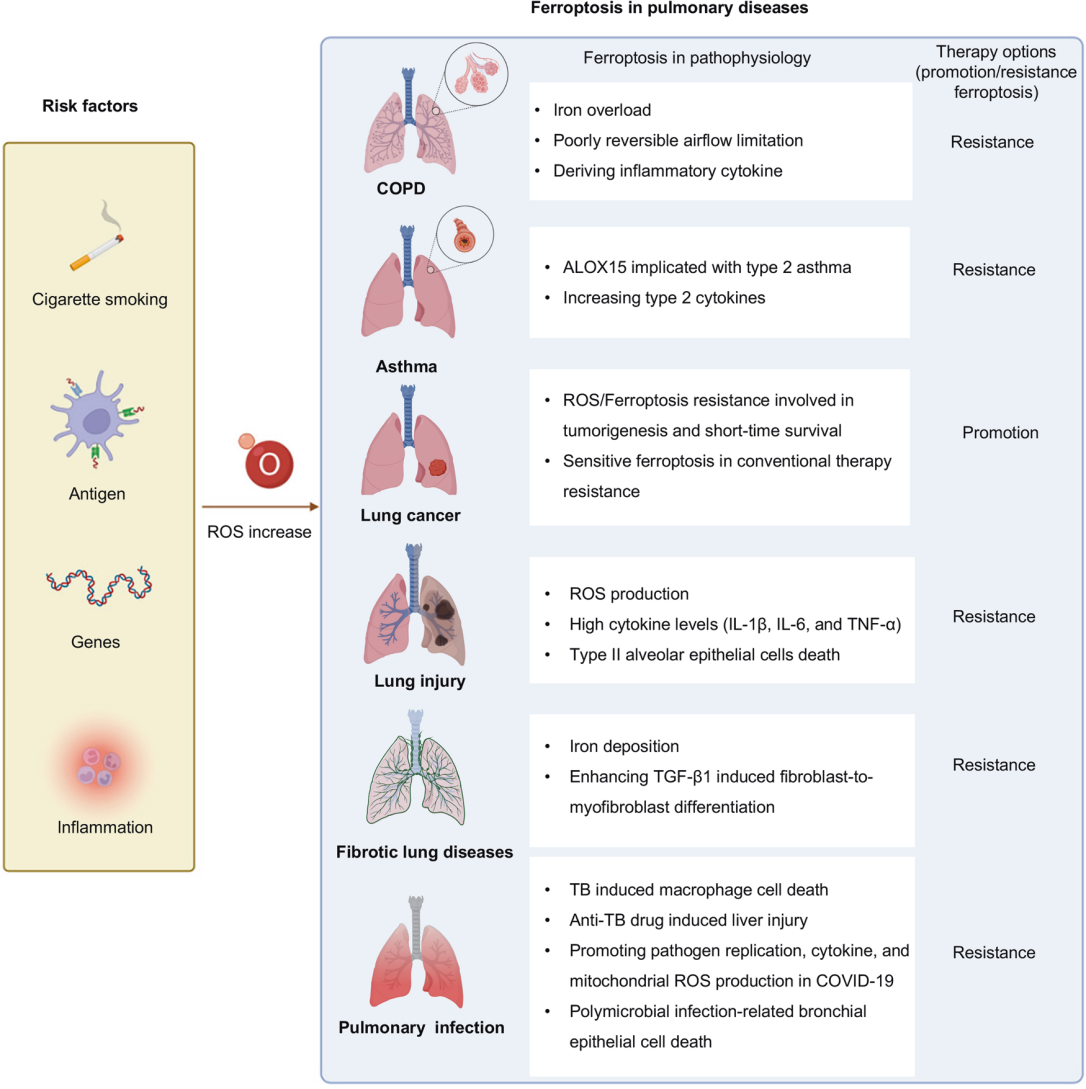
Role of ferroptosis in pulmonary diseases. Risk factors to induce ROS generation in the pulmonary are a high oxygen environment because of its interface with the outside environment, smoking stimulation, genes, non-pathogenic or pathogenic antigen exposure, and antigen-induced inflammation. Ferroptosis plays a complex role in pulmonary diseases such as COPD, asthma, lung cancer, lung injury, fibrotic lung diseases, and pulmonary infection. The pathogenic roles for ferroptosis have been proposed in these diseases. Anti-/pro-ferroptosis therapy will offer more options.

The altered iron or redox homeostasis and lipid peroxidation have been shown in pulmonary diseases in vivo or in vitro. Therefore, understanding the relationship between ferroptosis and pulmonary diseases helps to confirm whether pro/resistance ferroptosis is more efficacious than traditional healing methods. In this section, how ferroptosis participate in pulmonary diseases models and patients will be described in detail as follows (Fig. 4), and related promising medicine is listed in Table 3.

**Table 3:** Selected therapeutic approaches for targeting the ferroptosis pathway in pulmonary disease.

Target/agent	Function	Disease	References
GCL inhibitor			
Buthionine sulfoximine	Depletion of GSH, enhancing cytotoxicity, improving efficacy of combination therapy	Lung cancer	[161, 168]
GSH inhibitor			
Cisplatin	Widely used in different stages of NSCLC and SCLC with GSH consumption and formation of DNA interstrand cross-links to impair DNA function	Lung cancer	[169–171]
SLC7A11 inhibitor			
Sulfasalazine	Inhibiting system xc^-^ in invasiveness and drug resistance NSCLC cells	Lung cancer	[172]
HG162	Dose-dependently inhibiting cystine taken and GSH level in KRAS-mutant LUAD cells		[173]
Iron activators			
Salinomycin	Blocking iron translocation, inducing an iron depletion, leading to lysosomal degradation of ferritin and ferroptosis, reversing cancer stem-like cell features	lung cancer	[174, 175]
Erianin	Inducing lung cancer cell ferroptosis through iron uptake	lung cancer	[176]
Iron chelator			
Deferoxamine	Reducing iron levels and protecting ferroptosis	Fibrotic lung diseases; Pulmonary infection	[142, 145, 177]
Vitamin E (α-tocopherol) Coenzyme Q_10_ Ferrostatin-1 Liproxstatin-1	Radical-trapping antioxidant even when GPX4 deletion	COPD; Fibrotic Lung Diseases; Lung injury	[131, 145, 178, 179]

*GCL* gamma-glutamylcysteine ligase, *LUAD* lung adenocarcinoma, *GSH* glutathione, *GPX4* glutathione peroxidase 4, *COPD* chronic obstructive pulmonary disease.

### Chronic obstructive pulmonary disease (COPD)

COPD is an airway limitation disease, with the remodeling of the small-airway compartment and elastic recoil lost by the emphysematous destruction of parenchyma, and the pulmonary function shows forced expiratory volume in 1 s (FEV1) decline [80, 81]. Lung development, genetic abnormalities, and smoking stimulation are the main causes of COPD. Especially, smoking is a well-established risk factor for COPD among them [82, 83].

Under the stimulation of CS, macrophages, neutrophils, and lymphocytes are recruited in the small airways and the lung parenchyma to secret inflammatory factors to induce oxidative stress and pulmonary epithelial cells death [84, 85]. Among them, the accumulation of monocyte-derived macrophages increases leukotriene B4 (LTB4) secretion and triggers the expression of ACSL4 on epithelial cells, thereby inducing alveolar epithelial type 2 (AT2) cell ferroptosis [85]. In addition, COPD patients are companied with iron homeostasis imbalance and lipid peroxidation. Under CS exposure, the NOCA4-mediated ferritinophagy pathway and IRP2 upregulated mitochondrial iron overload are initiated, whereas GPX4 related defending pathway is inhibited [85–87].

Genome-wide association studies (GWAS) revealed that IRP2 is a susceptibility gene to COPD [88]. Lung epithelial cells with higher IRP2 expressions are accompanied by increased secretion of airway mucus, elevated airway remodeling, dysregulated infiltrated immune cells expressing inflammatory mediators (IL-33, IL-6), which contribute to the severity of acute COPD exacerbations secretion, and declined FEV1 in CS-induced COPD mice [86, 89, 90]. In the meanwhile, supplementation of mitochondrial iron chelator or fed with a low-iron diet protects them from CS-induced COPD.

In short, the change of iron homeostasis and ferroptosis induced by CS plays an important role in COPD occurrence. Targeting iron hemostasis and lipid peroxidation may be an optional treatment for COPD.

### Asthma

Type 2 high asthma accounts for 40–70% of asthmatics, which is an airway inflammation disease with elevated type 2 cytokines (such as IL-4, IL-13, IL-5) [91]. ALOX15 is a proinflammatory event in asthma. Type 2 cytokines are inducers of ALOX15 expression [92], which in turn supports the release of chemokines to recruit activated T cells in lung epithelial cells in vitro [93]. The elevated expression of ALOX15 in the bronchial epithelium or eosinophils of BALF in both childhood and adult asthmatics is associated with allergen sensitization and airway inflammation [94, 95]. A scaffold protein inhibitor of protein kinase cascades phosphatidylethanolamine-binding protein 1 (PEBP1) complexes with 15-LOX isoforms, 15LO-1, and 15LO-2, and the number of co-localized PEBP1/15LO-1 puncta in the freshly brushed airway epithelial cells have a strong correlation with increased FeNO in type 2 asthma patients, implying the potential role of ferroptosis in asthma [96].

### Lung cancer

Lung cancer, including non–small-cell lung cancer (NSCLC) and small-cell lung cancer (SCLC), is the leading cancer-related mortality worldwide [97]. NSCLC is the most common type of lung cancer accounting for 76% [98, 99], mainly containing LUAD and lung squamous cell carcinoma (LSCC). SCLC accounts for ~15% of lung cancers with a strong predilection for early metastasis and poor prognosis [99].

#### NSCLC

Exposed to a high oxygen environment, metastatic or primary NSCLC selectively expression of NFS1, which is a cysteine desulfurase enzyme critical for ISC biosynthesis by harvesting sulfur from cysteine [100]. In LUAD, NFS1 staining is significantly higher in situ compared with poorly differentiated and high-grade regions [100]. The suppression of NFS1 alone does not impair the expression of GSH or increase ROS to induce ferroptosis. However, suppression of NFS1 enhances the sensitivity of ferroptosis by activating the iron-starvation response in high oxygen tension [101]. Inhibiting iron overload is a major way to escape from ferroptosis for cancer cells. Deubiquitinase USP35, which directly binds with FPN and decreases the ubiquitinated level of FPN, stabilizes FPN to prevent iron overload and ferroptosis in lung cancer cells. Knockdown of USP35 enhances the sensitivity to chemotherapy-induced cell death [102].

SLC7A11 is highly expressed in lung cancer [103, 104] and is regulated by both transcriptional and translational pathways. SOX2, which contributes to the development of LSCC [105], is proven to promote the expression of SLC7A11 to maintain the stemness and ferroptosis resistance in cancer stem-like cells (CSLCs) as a transcription factor [104]. The expression levels of SLC7A11 and SOX2 are positively correlated in human LSCC [104]. In addition, RBMS1, directly interacting with translation initiation factor eIF3d [106], promotes lung cancer progression through translational regulating SLC7A11. Depletion of RBMS1 sensitizes ionizing radiation (IR) resistant lung cancer cells to ferroptosis [106].

The high expression of SLC7A11 in NSCLC survives less time due to GSH synthetic and ferroptosis resistance [107], suggesting that targeting xCT-mediated cysteine uptake would be a potential therapy for NSCLC. However, in some cases, cystine starvation does not improve the prognosis of NSCLC. NSCLC cell lines carrying high levels of GCLC directly generate g-Glutamyl-Peptide to reduce the glutamate-induced ROS level and ferroptosis under cystine starvation [108]. The generation of g-Glutamyl-Peptide via GCLC is regulated by the KEAP1-NRF2 pathway [108], indicating a potential treatment for NSCLC by targeting the KEAP1-NRF2 pathway. Moreover, around 16% of NSCLC patients are KEAP1 mutant and resistant to standard-of-care therapies including radiotherapy. A most recent work reveals that FSP1 is upregulated through NRF2-mediated transcription in KEAP1 mutant or deficient lung cancer cells. Targeting the CoQ-FSP1 axis renders KEAP1 deficient or mutation lung cancer cells sensitive to radiotherapy-induced ferroptosis [109].

Oncogenic mutation, especially epidermal growth factor receptor (EGFR) mutation is a major target in NSCLC patients. Although the objective response rate for the treatment of EGFR tyrosine kinase inhibitor (EGFR-TKI) is >70% [110], therapeutic resistance is inevitable. Nevertheless, activated EGFR cells are more sensitive to ferroptosis through activation of the MAPK pathway under cystine depletion. As MAPK pathway reduces expression of GPX4 and increases hydrogen peroxide production [111], thereby promoting EGFR-TKI resistant cells sensitive to ferroptosis inducers (erastin, RSL3) [112]. These findings indicate that ferroptosis-related genes may act as a prognosis prediction and a target bypass of the conventional treatment.

#### SCLC

SCLC is divided into neuroendocrine (NE) and non-NE SCLC marked by the loss of neuroendocrine features. Although non-NE SCLC is associated with the resistance to conventional chemotherapy [113], it is vulnerable to ferroptosis because of elevated LPCAT3 and ACSL4 mediated lipid remodeling. Separation of the subtype of SCLC and combined treatment to consideration of the role of ferroptosis in the plasticity of SCLC would be beneficial for improving overall survival [103].

#### Therapy for lung cancer

Emerging research shows that ferroptosis is a viable treatment option for lung cancer, especially in therapy-resistant lung cancer [114, 115]. Platinum is widely used in the treatment of lung cancer due to its GSH consumption and impaired normal DNA function (Table 3). In addition, chemo-resistance of platinum is Wnt-NRF2 pathway activated, GPX4 expression, and high consumption of GSH, all of which result in ferroptosis susceptible [116]. Targeting ferroptosis significantly enhances chemosensitivity in lung cancer. Moreover, the diagnosis and ferroptosis-targeted treatment of lung cancer via nano-particles have also made some progress. Prussian blue/calcium peroxide nanocomposites promote iron mineralization in lung cancers, which greatly facilitates early diagnosis of lung carcinoma and activates ferroptosis to inhibit tumor growth [117]. Most recently, a self-assembled pH-sensitive superparamagnetic iron oxide nanoclusters (SPIONCs) is reported to enhance in situ ferroptosis and apoptosis of lung tumors with radiotherapy and chemodynamic therapy via releasing iron in the tumor microenvironment (TME) [118].

Immune-checkpoint inhibitors (ICIs), especially therapeutic antibodies targeting PD-1/PD-L1, have been approved as efficient therapeutic regimens for lung cancer. ICIs activate the effector function of cytotoxic T-cell-driven antitumor response to release interferon gamma (IFNγ), which induces ferroptosis in tumor cells [119]. IFNγ downregulates the expression of SLC7A11 and SLC3A2 in tumor cells to inhibit cystine uptake. On the other hand, IFNγ directly initiate arachidonic acid-induced tumor cell ferroptosis via activating ACSL4 [120, 121]. Moreover, zero-valent-iron nanoparticle (ZVI-NP), which is used in the preclinical model, induces lipid peroxidation to initiate ferroptosis in lung cancer cells and augments antitumor immunity via eliciting the immunostimulatory TME [122].

### Lung injury

#### Acute lung injury (ALI)

Ferroptosis is not only associated with heart, brain, kidney, and liver injury but also involved in the pathogenesis of ALI induced by ischemia/reperfusion (I/R) or lipopolysaccharide (LPS)-mediated sepsis [123]. Recent findings clarify that increased expression of NRF2 exerts significant resistance to lipid peroxidation-induced injury in I/R by upregulating anti-ferroptosis genes (GPX4, SlC7A11) [124, 125]. Furthermore, electroacupuncture or treatment with metabolites such as obacunone and itaconate induces activation of NRF2 pathway to defend LPS-mediated ALI model [126–128]. Moreover, The AU-rich element (ARE)-binding factor 1 (AUF1), which acts as a switch for sepsis shock, suppresses ferroptosis by upregulating NRF2 and downregulating ATF3. AUF1 Knockout mice survived less time in sepsis-induced ALI model and exhibited severe lung injuries [129]. Decreased expression of GPX4 and GSH, increased production of MDA, and characteristic mitochondrial morphological changes of ferroptosis are exhibited in the ALI mice [130]. Erastin treatment further increases exacerbate edema, atelectasis, necrosis, inflammation, and fibrosis of pulmonary in the I/R mice, which is remarkably reversed by liprostatin-1 [125]. Furthermore, the injection of iron or Ferrostatin-1 via the tail vein respectively exacerbates or palliates lung injury and pulmonary edema [131]. Together, these findings suggest that inhibiting ferroptosis is a potential treatment for I/R or LPS-induced ALI.

#### Radiation-induced lung injury (RILI)

Radiotherapy is an important approach in the treatment of lung cancer along with a 5–20% incidence rate of RILI (including pneumonitis and pulmonary fibrosis) after thoracic radiotherapy [132]. Radiation-induced oxidative stress leads to the accumulation of inflammatory cells to secret cytokines facilitating the occurrence of RILI [133]. Radiotherapy decreases the expression of System Xc- and GPX4 in turn resulting in lipid peroxidation. This process indicates that radiotherapy activates lung cells to undergo ferroptosis and induce RILI. Moreover, ferroptosis inducers have a synergistic effect with radiotherapy whereas ferroptosis inhibitor mitigates pathologic changes of RILI [134, 135].

### Fibrotic lung diseases

Interstitial lung diseases (ILDs) are characterized as infiltrated interstitial inflammatory cells, cellular proliferation, fibrosis within the alveolar wall [136], in which interstitial fibrosis is the predominant phenotype [137]. There are a variety of causes to induce pulmonary fibrosis, such as different primary diseases (connective-tissue disease (CTD), sarcoidosis, Langerhans-cell granulomatosis, eosinophilic pneumonia, and pulmonary alveolar proteinosis), environmental exposures (inhalation of inorganic substances or organic particles), drugs, illicit drugs, or irradiation, and unknown reasons (idiopathic pulmonary fibrosis (IPF)). The commonly fibrotic ILDs are sarcoidosis, CTD-associated ILDs, and IPF [138].

Transforming growth factor-β1 (TGF-β1) induced fibroblast-to-myofibroblast differentiation is critical for pathogenesis and development of pulmonary fibrosis [139]. Ferroptosis inducer erastin enhances TGF-β1 induced fibroblast-to-myofibroblast differentiation pulmonary fibrosis models in vitro by inhibiting the expression of GPX4 and increasing lipid peroxidation, which is rescued by Fer-1 in HFL1 cell [140]. Additionally, liprostatin-1 inhibits collagen deposition and attenuates RILF through the NRF2 signaling pathway to repress the expression of TGF-β1 [141]. Moreover, iron deposition is found in pulmonary fibrosis patients and bleomycin-induced pulmonary fibrosis mice, whereas DFO protects alveolar epithelial cells against bleomycin-induced iron deposition and ferroptosis in vivo [142]. In addition, some ferroptosis-related genes (SLC40A1, NRAS et al.) in BALF of IPF patients serve as prognostic biomarkers [143, 144].

Paraquat (PQ), a popular poisoning substance, induces pulmonary fibrosis. The pathophysiology of PQ toxicity is oxidant/antioxidant imbalance-induced lipid peroxidation, which contributes to ferroptosis [145]. For the treatment of PQ poisoning, the ferroptosis inhibitors (DFO, Fer-1, Vitamin E et al.) would be potential novel treatment strategies [145].

### Pulmonary infection

#### Tuberculosis (TB)

TB is concerned with the Mycobacterium tuberculosis (Mtb) infection. Inhaling Mtb is phagocytized by alveolar macrophages to limit the proliferation of Mtb, when the bacteria reach the lung [146]. Cell death of macrophages facilitating Mtb spread is a host detrimental process [147]. Mtb-induced macrophage necrosis is associated with ferroptosis including reduced GPX4, increased free iron, and lipid peroxidation in mice models. Notably, bacterial infection exhibits a marked reduction upon supplementation of Fer-1 in Mtb-infected mice [148]. Besides, perturbed iron homeostasis is a risk factor for tuberculosis progression and is used to diagnose tuberculosis [149]. Ferritin deficiency-induced iron overload consequently promotes Mtb growth, dissemination, and host death via accumulated lipid peroxidation and ferroptosis of macrophages in Mtb-infected mice [150, 151].

Isoniazid (INH) and rifampicin (RFP) used in combination are the first-line anti-TB regimen and metabolized in the liver accompanied by liver toxicity with large consumption of intracellular GSH, leading lipid peroxidation and hepatocyte death [152]. The phenotypes of increased iron concentration, lipid peroxidation, inactivation of GPX4, and upregulation of ACSL4 are observed in anti-TB drug-induced liver injury mice models [153]. Therefore, anti-ferroptosis is a possible approach for the therapeutic target of TB or to reduce anti-TB drug-induced liver injury.

#### Coronavirus disease 2019 (COVID-19)

COVID-19 is a highly contagious infectious disease by infection with a novel beta coronavirus severe acute respiratory syndrome coronavirus 2 (SARS-CoV-2) [154]. SARS-CoV-2 attacks human cells at multiple points, in which the lung and the throat are the main targets [155]. Among COVID-19 patients, 14% have the severe disease [156]. The rapid progression of respiratory failure soon meets the criteria for acute respiratory distress syndrome (ARDS), which is the primary driver of mortality in COVID-19 [157, 158].

Accumulation of oxidized PE species in BALF and increased expression of TFR in lung tissue in Syrian hamsters with ALI induced by SARS-CoV-2 infection reveals that SARS-CoA-2 induces ferroptosis [159]. 4-HNE is positively stained in myocardial tissue and the proximal tubules in a severe COVID-19 lethal cardiogenic shock male patient [160]. In addition, SARS-CoV-2 significantly suppresses the expression of GPX4 mRNA in Vero cells [161]. Macrophages and monocytes are the most enriched immune cell types in the lung of COVID-19 patients [162]. Monocytes promote SARS-CoV-2 replication and cytokine (HIF-1α) expression, enhance glycolysis and trigger mitochondrial ROS production under elevated glucose levels in obese/diabetic COVID-19 patients. Under this circumstance, the monocytes promote epithelial cell death with the secretion of HIF-1α in vitro [163]. These findings indicate that the compounds targeting ferroptosis are potential candidates for COVID-19.

#### Pseudomonas aeruginosa

Polymicrobial infection prefers to occur in the respiratory tract of cystic fibrosis (CF) patients in which *Pseudomonas aeruginosa* is a major frequently cultured CF bacterial pathogen [164]. *Pseudomonas aeruginosa*, which contains ALOX15 (referred to as pLoxA) induces ferroptosis in human bronchial epithelial (HBE) cells through selectively oxidation of membrane PLs (particularly PE-containing AA-CoA) [165]. Although *Pseudomonas aeruginosa* possesses ALOX15, it lacks PUFAs-lipid substrates for ALOX15 [166]. *Pseudomonas aeruginosa* secretes vesicles containing pLoxA as a pathogenic strategy of delivering pLoxA into host cells to induce lipid peroxidation. Together, targeting pLoxA is a promising therapy in the treatment of *Pseudomonas aeruginosa-induced* respiratory tract infections to a large extent to overcome its antibiotic resistance.

## Conclusions and future perspectives

Oxidative stress is a common phenomenon under oxygen, determining cell fate by the response to oxidative stress. Oxidative modification of lipids in membrane bilayers induced lipid peroxidation is a widespread consequence of oxidative stress [14]. Here, we summarized the regulation pathway of ferroptosis and the relationship between ferroptosis and pulmonary diseases. Although targeting ferroptosis is effective in experimental mice of pulmonary diseases models, many questions still need further clarification.

The ferroptosis inducers such as IKE and ML162 mainly suppress tumor growth by inhibiting SLC7A11 or GPX4. Targeting lung cancer cells harboring high levels of SLC7A11 or GPX4 via ferroptosis inducers could be a potential treatment. However, some lung cancer cells express low levels of SLC7A11 or GPX4. Therefore, it is basilic to screen specific genes as markers and targets to obtain greater clinical benefits. In addition, the uptake of PUFAs and antioxidants like Vitamin E from the diet are closely related to ferroptosis, so dietary management in targeted ferroptosis is considerable. Furthermore, accurately assessing oxidative stress and metabolic changes in lesions before the ferroptosis-related drugs chosen in patients with comorbidities need to be further investigated. Although system ferritin increasement is related to inflammatory states, such as malignancy, infection, and autoimmune diseases [167], the indices of systemic iron metabolism in ferroptosis-targeted therapy are uncertain.

At present, the role of ferroptosis in lung diseases is mostly preclinical evidence, a series of evaluation criteria should be developed before clinical application. As ineluctable ferroptosis is tightly associated with pulmonary diseases, further ferroptosis-related research in pulmonary diseases acquires more therapy choices and biological symbols. Targeting ferroptosis as an adjunctive therapy choice to improve operative outcomes is necessary.

## Data Availability

All the data supporting the findings of this study are available from the corresponding author on reasonable request.
